# COVID-19: Learning from Lessons To Guide Treatment and Prevention Interventions

**DOI:** 10.1128/mSphere.00317-20

**Published:** 2020-05-13

**Authors:** Chris R. Triggle, Devendra Bansal, Elmoubasher Abu Baker Abd Farag, Hong Ding, Ali A. Sultan

**Affiliations:** a Department of Pharmacology, Weill Cornell Medicine-Qatar, Doha, Qatar; b Ministry of Public Health, Doha, Qatar; c Department of Microbiology and Immunology, Weill Cornell Medicine, Cornell University, Doha, Qatar; National Institute of Allergy and Infectious Diseases

**Keywords:** COVID-19, drugs, vaccines

## Abstract

Coronavirus disease 2019 (COVID-19) is caused by the novel coronavirus severe acute respiratory syndrome coronavirus 2 (SARS-CoV-2) and first emerged in December 2019 in Wuhan, Hubei province, China. Since then, the virus has rapidly spread to many countries. While the outbreak in China appears to be in decline, the disease has spread across the world, with a daily increase in the number of confirmed cases and infection-related deaths. Here, we highlight (i) the lessons that have been learnt so far and how they will benefit reducing the impact of COVID-19 disease and (ii) an update on the status of drug treatment and vaccine development to prevent COVID-19 and potential future related pandemics. Although the mortality rate is clearly higher than for influenza, the rate does seem to vary from country to country, possibly reflecting differences in how rapidly local health authorities respond to isolate and effectively care for the affected population. Drugs are urgently needed for both prophylaxis and the treatment of severely ill patients; however, no proven effective therapies for SARS-CoV-2 currently exist. A number of drugs that have been approved for other diseases are being tested for the treatment of COVID-19 patients, but there is an absence of data from appropriately designed clinical trials showing that these drugs, either alone or in combination, will prove effective. There is also a global urgency to develop a vaccine against COVID-19, but development and appropriate testing will take at least a year before such a vaccine will be globally available. This review summarizes the lessons learnt so far from the COVID-19 pandemic, examines the evidence regarding the drugs that are being tested for the treatment of COVID19, and describes the progress made in efforts to develop an effective vaccine.

## Abstract (precis)

Coronavirus disease 2019 (COVID-19) is caused by the novel coronavirus severe acute respiratory syndrome coronavirus 2 (SARS-CoV-2) and first emerged in December 2019 in Wuhan, Hubei province, China. Since then, the virus has rapidly spread to many countries. While the outbreak in China appears to be in decline, the disease has spread across the world, with a daily increase in the number of confirmed cases and infection-related deaths. Here, we highlight (i) the lessons that have been learnt so far and how they will benefit reducing the impact of COVID-19 disease and (ii) an update on the status of drug treatment and vaccine development to prevent COVID-19 and potential future related pandemics.

## INTRODUCTION

The coronavirus (CoV) now named severe acute respiratory syndrome coronavirus 2 (SARS-CoV-2) is responsible for the disease coronavirus disease 2019 (COVID-19) and was first detected in early December 2019, in Wuhan City, Hubei Province, China. The disease was initially described as a “pneumonia of unknown etiology” with high fever that was not responding to drug treatment. The early cases were linked to the Huanan seafood market, and by the end of December, >25 similar cases had been reported. Unfortunately, unlike earlier cases of avian flu, the outbreak did not peter out, and it was on 31 December 2019 that the World Health Organization (WHO) was informed and cases were finally reported and entered into the Chinese National Health Database. SARS-CoV-2 is a zoonotic virus related to the severe acute respiratory syndrome coronavirus (SARS-CoV) that was responsible for a 2002 outbreak and is also related to Middle East respiratory syndrome coronavirus (MERS-CoV), responsible for Middle East respiratory syndrome (1). COVID-19 subsequently has, with the exception of Antarctica, spread globally, and the number of cases is growing daily (2–4). Although, as a result of the 2002 SARS-CoV epidemic, China had created a rapid-response infectious disease reporting system, there was an unfortunate delay in recognizing the early cases in December 2019 as a new SARS-like outbreak and there was a rapid spread of COVID-19 in Hubei and beyond (5).

The incubation period for COVID-19 ranges from 2 to 14 days (6, 7). Up to 44% of COVID-19 cases spread from person to person before symptoms appear, thus emphasizing the difficulty in containing the spread and the importance of testing for the virus as well as the importance of control measures to reduce the spread of the virus such as hand washing, social distancing, and the use of face masks (8). Furthermore, some people are initially asymptomatic and may remain asymptomatic and serve to spread infection throughout the community, although the report from China stated that the majority of those tested and initially found asymptomatic went on to develop COVID-19 (9).

The clinical manifestations represent a wide spectrum of disease ranging from mild to severe respiratory syndrome influenza-like illness with mainly lower respiratory tract symptoms, complicated by pneumonia and acute respiratory distress syndrome (ARDS), high fever, and headache (10). In many cases, loss of taste and smell and severe gastrointestinal symptoms are reported, as are cardiac problems, with the latter being perhaps secondary to a cytokine storm such as is seen in the more severely affected patients (10–12). Based on the data from China, approximately 80% of people infected with COVID-19 present with mild to moderate disease that may include pneumonia; about 14% have severe disease with blood oxygen saturation (≤93%); and 6% are critical, with respiratory failure, septic shock, and/or serious multiple-organ dysfunction or failure (9).

Elderly persons and those with multiple comorbidities such as cancer, cardiovascular disease, diabetes, and immunosuppressive diseases are at a higher risk of acquiring the infection and developing complications and are more likely to succumb to the infection (13, 14). The case fatality rate is estimated to range from 2% to 5%, but with country-to-country variability and likely inaccurate data reported in many countries, the true number of infected persons is unknown due to inadequate testing (2, 3, 6).

From a public health perspective, it is important that health care workers and the public are aware of the resilience of the virus. As reported in the *New England Journal of Medicine*, when in the aerosol form as it leaves a human host, the SARS-CoV-2 virus has a half-life of 1.1 to 1.2 h, a range very similar to that seen with SARS-CoV-1 (15). However, the half-life of SARS-CoV-2 on metal and plastic is much longer, with a half-life of 5.8 h for stainless steel (and less for copper and cardboard) but a half-life of 6.8 h on plastic. Thus, appropriate care needs to be taken in the handling and cleaning with disinfectant of potentially virus-contaminated packages and containers (15). In addition, because of the potential presence of asymptomatic carriers of SARS-CoV-2 in the community, the experiences from the COVID-19 pandemic point very clearly to the need to impose social distancing and for the wearing of face masks and the cancellation of public events, including sports and religious gatherings, thus limiting and slowing the spread of a pandemic.

In this article, we discuss the current status, treatment, and prevention of COVID-19 and the lessons that have so far been learnt and that should therefore help the world to prepare for any future pandemics.

## 

### A global pandemic: what lessons have been learnt?

During the first 4 months of 2020, there was a very rapid rise in the number of cases of COVID-19 and a rapid global spread from the first case officially confirmed in Wuhan on 31 December 2019 to cases being reported in several other countries in Asia, Europe, and North America and in Australia and New Zealand before the end of January 2020. COVID-19 was first reported in the Middle East in February 2020 and in South America, Africa, and South Pacific countries in March. By 5 May 2020, more than 3.7 million cases of COVID-19, more than 250,000 deaths, and more than 1.2 million recoveries had been reported (2–4).

In the absence of a vaccine or drugs with known effectiveness against the SARS-CoV-2 coronavirus, there was a highly variable response to the threat of COVID-19. China, South Korea, Taiwan, and Vietnam all offer success stories in the control of the spread of this disease. The main lesson from their experiences is that aggressive and early action is critically important. China rolled out the most ambitious, agile, and aggressive disease containment effort in history and, although experiencing >82,000 cases and >4,600 deaths to date, seems to have controlled the spread of COVID-19, although China is now pursuing an equally vigorous regimen to prevent a second wave of infections as a result of citizens returning from overseas. The strategy that underpinned this containment effort was initially a national approach that promoted universal temperature monitoring, masking, and hand washing (9). On 23 January 2020, the Chinese government announced a lockdown in the city of Wuhan (population, 11 million) (9, 16). At that point, the total number of known cases in China was about 1,000. Soon the lockdown was expanded to about 15 other cities, covering the whole Hubei province with a population of about 57 million. The WHO described the actions taken by China as “unprecedented in public health history.”

Taiwan, based on the country’s experience with the earlier SARS-CoV outbreak, established the Taiwanese National Health Command Center in 2004 and was well prepared to contain the spread of COVID-19. Immediately after the WHO was informed of the COVID-19 outbreak on 31 December 2019, Taiwan officials began checking all incoming airline passengers from Wuhan with proactive testing and, as appropriate, quarantine (17). As of 5 May 2020, Taiwan, with a population of approximately 25 million, has reported <450 cases and only 6 deaths (2, 3).

Similarly, South Korea took aggressive action manifested in the early testing of hundreds of thousands of its people and tracking and isolation of infected individuals, even using smartphone and GPS technology (18). Similar approaches have been considered in the past, such as in the United Kingdom with FluPhone, but, to date, there has been reluctance or disinterest with respect to accepting what might be considered a breach of privacy (18). South Korea also introduced drive-through testing for COVID-19 and achieved rates of testing of approximately 10,000/day, representing an approach that is envied and is beginning to be copied by the rest of the world. As a result, in South Korea the first wave of COVID-19 infection is now receding and the situation seems to be under control. Based on the experiences in China and South Korea, it can be argued that government action that may initially be seen as excessive, unjustified, and possibly overly invasive of individual rights is essential. Thus, such stringent controls have benefits in controlling the spread of a pandemic and reducing mortality.

Vietnam is an example of a country with limited resources that was proactive in responding to COVID-19, and, as in the case of Taiwan, its Ministry of Health, hospitals, and clinics were well prepared before the country recorded its first case on January 23 2020. Authorities in Vietnam checked passengers at airports, restricted movement where necessary, followed up possible contacts, closed schools, and, as of 5 May 2020, had administered >200,000 tests for the virus and recorded only 271 cases (2, 3).

Among non-Asian countries, New Zealand stands out with a low number of cases that as of 5 May 2020 stood at <1,500 cases (308 per million capita), with only 20 deaths (2, 3). New Zealand was also proactive in checking incoming airline passengers, requiring returning citizens to self-quarantine, tracing contacts, and then finally locking down the country. Interestingly, the majority of the COVID-19 cases occurred in clusters of ∼200 individuals, suggesting that contact tracing may not have been completely effective.

In many other countries, COVID-19 spread very quickly with devastating effects. Iran has been one of the hardest hit by COVID-19 infections, and the failure to restrict travel at an early stage in the outbreak and lack of availability of hospital supplies due to the U.S.-initiated sanctions may have been contributing factors (2–4). In March, a number of countries in Europe—first Italy, next Spain, and then the United Kingdom—soon rivaled and surpassed China as the epicenters for COVID-19, only to be overtaken by the United States as cases skyrocketed there at the end of March 2020.

In Italy, the first case was identified in the Lombardy region of Northern Italy in early February 2020, and the infected individual had been a recent visitor to China; however, the second case was not confirmed until 20 February 2020 and that person had no prior contact with the first case and had not recently visited China. Many more cases were quickly confirmed, indicating that COVID-19 was already widely spread in the community. The availability of testing and the difficulties in containing the spread of the virus undoubtedly contributed to the rapid increase in confirmed cases and deaths. Although we do not have a clear answer as to why there was such a high case mortality rate in Italy (>13% as of 5 May 2020 [2, 3]), age may be one factor, as the citizens of Italy represent the second oldest population in the world (after Japan), with approximately 60% of the population over 40. Higher mortality in care homes may also be a factor, and this problem has been reported in several countries. Regardless, in a comparison of the age profiles of COVID-19 cases and deaths in Italy versus China, distinct differences have been noted, and although mortality increases with age, the case mortality rates are lower in the Chinese population (19). In China, the mean age of those who have died was reported to be 79.5 years, with most suffering from at least one comorbidity and with approximately 75% of those with COVID-19 disease being over 50 years old, whereas only 14 people under 50 died from the disease (16). A lesson can also be learnt from a community, Vò, in northern Italy, where aggressive contact tracing was used to seek out and identify asymptomatic infected individuals and where all ∼3,300 members of the community were tested, resulting in much better control of further infections (20).

Germany, despite having identified a high number of confirmed cases of COVID-19 of 5 May 2020, managed to keep its case-fatality rate very much lower than that seen in either Italy or Spain: ∼3.7% versus ∼10% to 13% (2, 3, 19). The answer would seem to have been a proactive testing program utilizing a large network of independent laboratories that started testing as early as January 2020. Other factors, including the number of available hospital beds and a lower population age than in Italy, may also have contributed (21).

In the United Kingdom, the government initially hoped that the development of herd immunity would reduce the impact of COVID-19. The British government also depended upon public cooperation to restrict travel, and it was not until 23 March 2020, and too late, that the Prime Minister announced that people would be allowed out of their homes only for “nonessential” reasons and that the police had the power to impose fines on offenders.

Similarly, in the United States, despite the first case being recorded in Washington state on 21 January 2020, there was an initial dismissal at the federal level of the severity and risks of COVID-19 and there were initial problems with the accuracy of CDC’s COVID-19 test kit. As a consequence, the infection rapidly spread, at least in part due to U.S. citizens returning (primarily from Europe) who were already infected (22).

A number of other factors might also contribute to the differences in infection and fatality statistics. Thus, based on genomic analysis of 103 genomes rom GenBank, two distinct strains of SARS-CoV-2 have been reported, with the L type more prevalent in the early stages of the breakout in Wuhan and also more aggressive than the evolutionary older S type (23). However, no supportive evidence has been produced indicating that such strain differences explain the differing country-to-country mortality rates. It has also been argued that seasonal flu is more prevalent during the winter months and wanes when warmer weather arrives. There is no evidence that weather would dramatically affect the spread of COVID-19, and, for example, countries such as Singapore have not been spared COVID-19, with the number of cases per million similar to the rate in Sweden (2, 3). Another factor may relate to differences in vaccination requirements from country to country; of particular interest is whether the Mycobacterium bovis BCG (Bacille Calmette Guérin) vaccine for tuberculosis provides protection against COVID-19. Thus, countries without a national requirement policy, or that have dropped the requirement, such as France, Italy, Spain, The Netherlands, and the United States, have had comparatively high infection and case fatality rates for COVID-19 whereas countries such as China and Japan that have a policy requiring vaccination of newborns report much lower levels (24). Whether there is a cause-and-effect relationship between different strains of SARS-CoV-2, weather, or BCG vaccination and infection and susceptibility to COVID-19 requires careful retrospective epidemiological evidence that will require further analysis. Finally, differences in the reporting of COVID-19 deaths may distort statistics, as has been evident with the apparent omission of those dying in care homes as well as deaths due to other morbidities that may have been attributed to COVID-19.

### How can we benefit from the lessons of COVID-19?

After the 2002 SARS-CoV epidemic, the world was sensitized to the necessity of a fast response to contain future zoonotic infections. In recognition of the threat, China established a nationwide Web-based automated system for reporting and responding to such infections (5). Unfortunately, COVID-19 still managed to catch the world flat-footed and the lack of preparedness in many countries rapidly became apparent. We can, however, better prepare for the next potentially equally lethal outbreak. Recommendations include the following:

1. Establishing a rapid reporting system such that any unusual infectious outbreak, e.g., “pneumonia of unknown origin”, is immediately reported to the national health organization for appropriate investigation, action, and notification of the WHO should be a high priority.
2. Immediately isolating the infected person(s) and identifying and quarantining individuals who have been in contact with infected persons is crucial.
3. When, as in the case of COVID-19 in Wuhan, spread of a virus has occurred within a local community, it is essential to initiate a rigorous attempt to identify all persons who might have been in contact with the infected individuals for appropriate testing and potential quarantine. An immediate lockdown of a town or area should also be considered. The rigorous pursuit of contacts is extremely important; as we have learnt with COVID-19, many infected individuals may not show symptoms of the disease and could become “super spreaders.”
4. If the spread of the infection is no longer localized, steps should be taken to impose social distancing, limit travel, limit public gatherings, including sporting and religious events, quarantine, and impose other actions to better contain the spread of the pathogen. The lessons that we have learnt from COVID-19 are that these actions require 100% cooperation from the public and strong endorsement and, if necessary, enforcement by governments and that these clearly have not been evident in all countries.
5. As was the case for COVID-19, the early genomic identification of the causative pathogen is important and can facilitate determining and developing the optimal treatment options.
6. Vaccine development could be enhanced by pursuing progress and clinical trials made with earlier vaccines developed, for instance, for SARS-CoV on the basis that such a vaccine(s) may also benefit patients infected by a related pathogen (coronavirus) or help facilitate the development of new vaccines.
7. Enhancing of drug development programs should be undertaken and would serve to identify and establish preclinical testing of candidate molecules effective against coronaviruses and other potential zoonotic viruses.
8. Establishing a global network with a mandate to ensure that there is sufficient personal protective equipment and hospital equipment available to all affected countries to deal with a pandemic should also be performed to constrain the global spread of a pandemic.

As discussed, Asian countries, such as China (including Hong Kong), South Korea, and Taiwan, have generally fared better than most European countries and the United States with respect to containing the spread of COVID-19 and this can be attributed in part to the proactive measures taken to control and proactively test for COVID-19. However, another significant difference between Western and Asian countries is the wearing of face masks, a practice that in some countries, for example, China, South Korea, and Japan, was already very common and almost ubiquitous, even in the absence of a known infection. In contrast, in the West it has been assumed that an individual wearing a face mask is infected, with that individual possibly subjected to harassment. Furthermore, in the West the argument has been made that there is an absence of evidence that wearing a non-N-95 surgical mask would provide protection against COVID-19 and that encouraging asymptomatic noninfected people to wear face masks would further strain the personal protective equipment supply train and reduce availability to health care personnel. However, as stressed above, it should be a priority for governments to ensure that adequate stocks of personal protective equipment are maintained and thus that such shortages should not occur. Interestingly, as correctly stated by Feng et al. (25) with reference to the benefits of wearing a face mask, “however, there is an essential distinction between absence of evidence and evidence of absence.” Indeed, proof has been provided that wearing a surgical mask can reduce droplet transmission from infected people (26). Of course, surgical masks are routinely worn by hospital personnel when treating patients, so an assumption can be made that they likely do reduce infections transmitted from patients to health care personnel and vice versa. Interestingly, many countries have reversed their recommendations on the wearing of face masks and now require their use. Undoubtedly, this is an appropriate decision, as it should reduce infection transmission of SARS-CoV-2 from that group of “silent” asymptomatic carriers to the noninfected public.

### Treatment.

On 10 January 2020, the SARS-CoV-2 genome sequenced by Chinese researchers was made available in GenBank by Yong-Zhen Zhang of the Shanghai Public Health Clinical Center & School of Public Health, Fudan University (27). SARS-CoV-2 is an RNA molecule with 29,891 bases encoding 9,860 amino acids and containing 15 genes, one of which is the S gene, which codes for the spike glycoprotein located on the surface of the viral envelope responsible for binding to the host’s cell via a receptor that for humans is angiotensin (Ang) converting enzyme 2 (ACE2) (27, 28). Of significance is that there is only 40% homology between SARS-CoV-2 and other SARS viruses for the external binding domain of the virus spike protein (17). This may be of importance with respect to assuming that drugs that proved at least potentially successful when used to treat SARS or MERS patients might not be as effective, or might be entirely ineffective, against SARS-CoV-2 (29).

Currently, no specific therapeutic agents and preventive vaccines are available and approved for the COVID-19. Furthermore, it is anticipated that, despite unprecedented research activity, a vaccine will not be available before 2021 (30). However, a number of drugs that have been approved for other diseases, some of which have been tried in patients with SARS-CoV and MERS-CoV, are being evaluated for the treatment of COVID-19. The drugs include remdesivir, baricitinib, chloroquine, hydroxychloroquine, the interleukin-6 (IL-6) receptor monoclonal antibody tocilizumab, and the anti-influenza drugs favipiravir and umifenovir (31, 32). The advantage of investigating these drugs is that there is already an extensive knowledge base with respect to their use and safety in humans; however, as is also the case for vaccines, it is essential that, despite the urgency, the introduction of new therapies should not be rushed at the expense of safety (33).

First, among the drugs being considered for use in treatment of COVID-19 is chloroquine, which was first synthesized by Bayer pharmaceuticals (Germany) in 1934 and has the advantage of being generic and inexpensive and shows good bioavailability when given orally. Chloroquine was first used in the 1940s to treat malaria and has also being successfully used against autoimmune diseases such as rheumatoid arthritis and lupus where the drug’s immunosuppressive effects against proinflammatory cytokines such as tumor necrosis factor alpha (TNF-α) and IL-6 are beneficial. Examples of the toxicity of chloroquine are well known, with central nervous system (CNS) side effects and macular retinopathy being the most serious; however, if chloroquine were to be used acutely for COVID-19 treatment, the risk of these side effects should be considerably less. Hydroxychloroquine is also effective against malaria, lupus, and rheumatoid arthritis, and its use for these diseases is not associated with significant cardiovascular side effects. Hydroxychloroquine also has the advantage that doses higher than those appropriate for chloroquine could be used for antiviral purposes. Nonetheless, concerns over cardiac toxicity should not be ignored, particularly if its use for COVID-19 is promoted before appropriate testing and if it is used in patients with preexisting cardiovascular disease. Both chloroquine and hydroxychloroquine do have direct inhibitory effects on cardiac sodium and potassium channels, albeit at high doses. The potential of cardiac toxicity is critical because evidence has emerged that SARS-CoV-2 may itself directly negatively affect cardiac function, perhaps as a result of a downregulation of ACE2 and/or secondary to a cytokine storm (12). Hydroxychloroquine has very good bioavailability, with a very long half-life of ∼22 to 30 days, and is metabolized via cytochrome P450s (CYP450). Azithromycin is a macrolide antibiotic that has been used in combination with hydroxychloroquine in several clinical trials. Azithromycin has CYP450-inhibitory effects and therefore may reduce the metabolism of hydroxychloroquine and also can precipitate cardiac arrhythmias via its ability to prolong the QT interval. Thus, a combination of the two drugs may result in an accumulation of hydroxychloroquine to toxic levels and trigger a fatal arrhythmia. In addition, there are serious concerns that, because hydroxychloroquine is also used for lupus and rheumatoid arthritis, diverting its use to COVID-19 may seriously affect the availability of the drug to patients being treated for these autoimmune diseases.

The mechanisms for the antiviral actions of chloroquine and hydroxychloroquine are not fully understood; however, it is known from *in vitro* studies that, as basic lysosomotropic drugs with a pK_a_ of ∼9.5, they become trapped in the acidic endosomes, alkalinizing the organelle, and thereby interfere with the pH-dependent entry of viruses, including coronaviruses, through the endolysosomal pathway (34). Chloroquine may also interfere with glycosylation of the cell membrane receptor for the coronavirus, ACE2, thus preventing the binding and cellular entry of the virus, as was shown *in vitro* for SARS-CoV (35, 36). A number of clinical trials are under way, and the early preliminary data from China demonstrated benefits in more than 100 patients with pneumonia where treatment with chloroquine resulted in elimination of the virus and improved recovery time compared to standard-of-care treatment (SOC) (37). Chloroquine is included in the 6th version of the Guidelines for the Prevention, Diagnosis, and Treatment of Novel Coronavirus-induced Pneumonia issued by the National Health Commission (NHC) of the People’s Republic of China. In another clinical trial, French researcher Didier Raoult in Marseille reported that a 6-day treatment with hydroxychloroquine plus azithromycin (with the antibiotic azithromycin included to reduce complications from secondary bacterial infections) performed initially with 25 patients and later with 80 patients with COVID-19 improved recovery and reduced viral load (38). However, these were small nonrandomized, unblinded trials and confirmative results are required from larger appropriately randomized controlled studies. One such randomized trial, DISCOVERY, has been launched in Europe and will compare the effectiveness of hydroxychloroquine plus standard of care versus standard of care alone and, in other arms of the study, the effectiveness of remdesivir, lopinavir plus ritonavir, and lopinavir plus ritonavir plus interferon beta (39). Interestingly, the results from a multicenter, open-label randomized trial of 150 patients in China treated with hydroxychloroquine versus SOC for 28 days did not demonstrate relief of symptoms and the hydroxychloroquine group reported significantly higher levels of side effects (30% versus 8.8%) (40). Treatment with hydroxychloroquine did, however, lower C-reactive protein (CRP) levels, thus supporting an anti-inflammatory action of the drug. Collectively and based on the current evidence, the available positive data are insufficient to demonstrate that treatment with hydroxychloroquine would benefit COVID-19 patients. Despite the current lack of conclusive positive medical evidence, COVID-19 patients are being treated with hydroxychloroquine in the United States under the provisions of the Emergency Use Authorization.

Second, baricitinib is a small-molecule Janus kinase (JAK) inhibitor that is currently approved for treatment of rheumatoid arthritis. Viruses, as mentioned above in the discussion of the putative antiviral effects of chloroquine, infect cells via cell surface receptor-mediated endocytosis, and for SARS-CoV-2 the receptor is ACE2. Endocytosis is regulated by kinases, including AP2-associated protein kinase 1 (AAK1), and baricitinib inhibits AAK1 (41). Arguably, baricitinib should disrupt ACE2-mediated SARS-CoV-2 endocytosis entry into cells within the same therapeutic plasma concentration range when used for rheumatoid arthritis, and therefore baricitinib is postulated to be another candidate for clinical trials to treat COVID-19 (41, 42).

Third, remdesivir was developed to treat infections due to the Ebola virus, but its use was not pursued. Remdesivir is a prodrug that is administered via intravenous (i.v.) infusion, and the active metabolite is an adenosine nucleoside analog that inhibits the action of viral RNA polymerase, thereby preventing viral replication. Remdesivir has been shown to be effective both *in vitro* and in a mouse model of SARS-CoV and appears to possess fairly broad anticoronavirus activity (43). Several clinical trials are under way in Asia, China, and the United States to study the efficacy and safety of remdesivir, including an NIH-sponsored randomized, controlled trial at the University of Nebraska Medical Center in Omaha. One small nonrandomized but multicenter trial of 61 patients with remdesivir reported clinical improvement in 36 of the 53 patients for whom data could be analyzed; however, there was no control group and a high number of side effects with serious side effects were reported in 12 patients (44).

Fourth, ritonavir and lopinavir are two protease inhibitors that have been widely used in HAART (highly active antiretroviral therapy) regimens as “ritonavir-boosted” protease inhibitor treatment of HIV. Ritonavir is not only a protease inhibitor but also an inhibitor of cytochrome P4503A4 and thereby reduces the metabolism and enhances and prolongs the action of the second protease inhibitor, in this case, lopinavir. Protease inhibitors prevent viral replication by virtue of blocking the proteolytic cleavage of precursor proteins that are required by the virus (45). The ritonavir/lopinavir combination has previously been shown to be effective *in vitro* and in an animal model of Middle East respiratory syndrome (MERS), and a clinical trial is under way to assess its effectiveness against MERS (46, 47). Furthermore, as reported in a case study in South Korea, a patient treated with ritonavir plus lopinavir displayed reduced viral loads and improved clinical symptoms; however, it was recognized that her recovery may simply reflect the natural course of the disease and regression to the norm (48). A randomized, controlled, open-label study of 199 COVID-19 patients at China’s Jin Yin-tan Hospital in Wuhan led to the conclusion that the combination of ritonavir plus lopinavir did not provide sufficient benefits over standard care, including reduction of viral RNA load, during the 28 days of the study (49). Further studies are needed; however, therapies directed at viral replication may prove to be more effective in the early stages of COVID-19 before significant pneumonia symptoms have developed.

Fifth, the anti-influenza drug favipiravir is an orally effective prodrug developed in Japan and approved in 2014 for the treatment of influenza. It differs from the neuraminidase inhibitors oseltamivir and zanamivir in that it inhibits the viral RNA-dependent RNA polymerase. In mid-March 2020, China announced that favipiravir had shown good clinical efficacy against COVID-19 (50). However, it remains to be clarified at what stage of infection with COVID-19 favipiravir is most effective. Another influenza drug that is being repurposed in Russia and China is umifenovir (often referred to by its brand name Arbidol), which is believed to prevent virus entry into the cell by inhibiting membrane fusion. Scientific support for the antiviral efficacy of umifenovir is sparse, however, and it has not been approved by the FDA for the treatment of influenza.

Another drug of interest is the antiparasitic drug ivermectin, where *in vitro* data suggest potential benefits; however, based on the pharmacokinetic evidence, the dose required to demonstrate *in vivo* efficacy for COVID-19 treatment in humans would most likely prove too toxic to use (51).

Camostat is a promising drug that is approved in Japan for pancreatitis and reflux esophagitis and targets the serine-threonine protease TMPRSS2. TMPRSS2 is the protease utilized by SARS-CoV-2 to prime the spike protein and facilitate binding to ACE2 and entry into the cell. Camostat has been reported to have inhibited SARS-CoV-2 entry into human epithelial cells in an *in vitro* assay, and clinical trials are being initiated (52).

Other approaches being pursued include the use of the cytokine interferon β, which can be applied by inhalation directly to the lungs, where it can activate the immune response. The Medicines and Healthcare Products Regulatory Agency (MHRA) and Health Research Authority (HRA) (53) have approved a fast-tracked phase-2 trial with interferon β in the United Kingdom. It is one of the drugs whose use, either alone or in combinations such as lopinavir/ritonavir, is being studied for treating coronavirus diseases (54). Serum therapy is also a consideration whereby the antibodies in the plasma of recovered COVID-19 patients, “human convalescent-phase serum,” are used to combat infection in severely infected patients, and such therapy has been used previously, for instance, during the 2009–2010 H1N1 influenza virus pandemic (55, 56).

In the most severely ill COVID-19 patients, a cytokine storm is a major contributor to the high mortality rate. Tocilizumab, the humanized monoclonal antibody against the soluble and membrane-bound forms of the interleukin-6 (IL-6) receptor, is being used with some success, but, as with the other drugs discussed here, larger trials are required with appropriate controls before science-based recommendations on use can be made (57).

Inhibitors of the renin-angiotensin-aldosterone system (RAAS) are used extensively in the treatment of cardiovascular disease and are beneficial to many patients with comorbidities of hypertension and diabetes and help protect renal function. ACE2 is ubiquitously expressed, with the highest levels detected in the lungs, cardiovascular system, kidneys, and the gastrointestinal system. ACE2 not only serves as a key receptor for the cellular entry of SARS-CoV-2 but also is an aminopeptidase that cleaves angiotensin I (Ang I) and angiotensin II (Ang II) into the Ang1 to Ang9 (Ang1–9) and Ang1–7 peptides. Critically, Ang1–7 signaling via the Mas receptor has cardiovascular protective activity, reducing levels of Ang II. Thus, loss of ACE2 would be expected to increase the risk to the patient, particularly in those with comorbidities. The epidemiological evidence for COVID-19 indicates that the elderly with comorbidities have a higher mortality rate, and many are likely already being treated with drugs that target RAAS for underlying cardiovascular diseases. Controversy has, however, arisen with regard to whether patients being treated specifically with angiotensin converting enzyme 1 inhibitors (ACEIs) or Ang II receptor 1 (blocker) inhibitors (ARBs) are protected, or are at higher risk, from COVID-19. ACE2 is downregulated in SARS-Co-V, and the loss of ACE2 may be a factor contributing to pulmonary dysfunction. Treatment with ACEIs and ARBs arguably, though without reproducible evidence, should increase ACE2 levels as a result of redirecting Ang 1 and Ang II to Ang 1–9 and Ang1–7, respectively, and should thus prove protective against COVID-19. Conversely, by increasing ACE2 activity, treatment with ACEIs or ARBs might enhance SARS-CoV-2 entry and exacerbate COVID-19 and lung pathology. The use of recombinant ACE2 protein has been proposed as a potential therapeutic approach to treat COVID-19, with the ACE2 protein acting as a decoy receptor for SARS-CoV-2 (58). The arguments both for and against the use of ACEIs and ARBs in the presence of COVID2 have been reviewed, leading to the conclusion that, based on the available evidence and given the risk reduction benefits of continued treatment with ACEIs and ARBs, there is no reason to switch treatment and that most patients should remain on their current therapy (59, 60). Further information will undoubtedly become available.

The contribution of traditional Chinese medicine (TCM) to the therapeutic management of SARS was evaluated as a result of its extensive use for SARS-CoV, and, although there was evidence of reduced morbidity and mortality possibly linked to anti-inflammatory effects, the design of the studies and the fact that treatment with TCM was often combined with Western medicines make it difficult to arrive at definitive conclusions with respect to the individual effectiveness of the TCMs (61). For SARS-CoV-2, it has been estimated that 85% of COVID-19 patients in China were treated with TCMs (62).

What is the prospect for new drugs specific for SARS-CoV-2? As previously noted, there is only 40% homology between the SARS-CoV-2 spike protein and that of other SARS viruses (29); thus, efforts should be directed at identifying lead molecules that specifically target the SARS-CoV-2 and the virus’s proteases. Mpro is one such target, and since it has no known human homologues, it should prove specific for the virus’s replication machinery (63).

### Symptomatic treatment.

Most patients with COVID-19 present with fever and many with headache. The drug of choice, if needed, for its antipyretic and analgesic actions is paracetamol (acetaminophen). Paracetamol is not a true nonsteroidal anti-inflammatory drug (NSAID) and lacks the anti-inflammatory, antiplatelet (anticoagulant) effects of NSAIDs and is also gastrointestinal sparing (gastro-sparing) and therefore avoids some of the potential problems that might be present in patients with comorbidities and who may also present with severe gastrointestinal symptoms of COVID-19 disease. Concerns have been presented by French officials with respect to the use of NSAIDs in patients with severe lung infections, such as those seen with COVID-19, as their anti-inflammatory actions may suppress the patient’s immune response. There is, as has been stated by the European Medicines Agency, currently “no scientific evidence” to support this argument (64). It should be noted that the use of both COX-2 selective drugs such as coxibs (celecoxib) and nonaspirin NSAIDs has been associated with an increase in cardiovascular risk; thus, the use of these drugs in patients with cardiovascular disease and COVID-19 should be evaluated on the basis of risk versus benefit (65).

### Vaccine prospective.

Currently, no vaccine is available to prevent infection with SARS-CoV-2. However, there are several lines of evidence suggesting that development of a vaccine against SARS-CoV-2 is achievable, including the following:

1. A group of researchers from Australia examined the immune response of a 47-year-old female who was experiencing mild-to-moderate symptoms of COVID-19 (66). They showed that recruitment of immune cell populations activated CD4^+^ and CD8^+^ T cells, together with IgM and IgG SARS-CoV-2-binding antibodies, in the patient’s blood before the resolution of symptoms. These data indicate that robust multifactorial immune responses can be elicited in response to SARS-CoV-2 infection (66).
2. A study involving four rhesus macaques found that contracting SARS-CoV-2 protected against future reinfections (67). When the researchers reinfected two of the four monkeys with the virus 28 days after the initial infection, a total of 96 nasopharyngeal and anal swabs tested negative after the reexposure of the monkeys to SARS-CoV-2. Data obtained following the euthanasia and necropsy of one of the two monkeys confirmed these results (67). These data suggest that the immune response developed by the 2 animals had protected them from future exposure to SARS-CoV-2.
3. Hoffmann and colleagues (52) studied whether antibodies made by people who had had a previous diagnosis of SARS would prevent SARS-CoV-2 virus entry into cells. They found that antibodies against the SARS-CoV S protein reduced the efficiency of infection into cells of a laboratory model virus with the SARS-CoV-2 spike (S) protein. They also observed similar results with antibodies against S proteins made in rabbits (68). These results indicate that neutralizing antibody responses raised against the virus S protein could offer some protection against SARS-CoV-2 infection, which may have implications for prevention of COVD19 infection.
4. Another study showed that antibody serum from four different mice could reduce by 90% infection with a laboratory model virus containing the SARS-CoV-2 S (69).
5. As argued by Casadevall and Pirofski (55), passive immunization with convalescent sera containing antibodies from patients who have recovered from COVID-19 could prevent COVID-19 infection. Evidence supporting the benefits of using convalescent plasma for COVID-19 has been provided by China and includes radiological resolution, reduction in viral loads, and improved survival (69).

Several efforts are under way to devise new vaccines, and phase 1 clinical trials are ongoing in the United Kingdom, the United States, Germany, France, and China. The first one is taking place at the Kaiser Permanente Washington Health Research Institute in Seattle, WA (70). In that trial, 45 healthy volunteers are to receive a vaccine that contains a segment of genetic code copied from SARS-CoV-2. As the vaccine does not contain the actual SARS-CoV-2, the participants will not develop COVID-19. At this stage, the main purpose of this trial is to confirm the safety of such vaccine and it might take 12 to 18 months before the vaccine can be deployed as a preventive vaccine against COVID-19 (71).

Moderna, Inc. (Cambridge, MA, USA), is developing the mRNA-based vaccine mRNA-1273, which codes for the appropriate coronavirus proteins. When this vaccine is injected into the body, immune cells in the lymph nodes process the mRNA and start immediately making the protein, which is then recognized and marked for destruction (71). Another mRNA-based vaccine is being developed by the CureVac Biotech Company in Tübingen, Germany, with plans to enter a human clinical trial in June 2020 (72).

In the United Kingdom, Sarah Gilbert and her colleagues at Oxford University have begun a human and animal trial of ChAdOx1 vaccine and expect it to be available by the end of the 2020 (73). In China, a recombinant vaccine has been developed by CanSino Biologics in collaboration with the Academy of Military Medical Sciences. A clinical trial has begun among volunteers between 18 and 60 years of age (74).

### Conclusion.

What is still unclear is the true mortality rate of COVID-19. What is clear is that the elderly with comorbidities such as cardiovascular disease, diabetes, cancer, and immunosuppression-related diseases are at greater risk; however, an unknown fact is what percentage of people with mild symptoms that perhaps were attributed to flu were misdiagnosed and therefore were omitted from being tested. Possibly the mortality will prove not to be high as has been reflected in some reports and hopefully will prove to be closer to 1%. This should become clearer in the coming months. It is also apparent that the overall global response to COVID-19 has been inadequate, with considerable differences in how rapidly and appropriately different nations have responded. These deficiencies emphasize the need for greater collaboration as well as the benefits of testing for the disease in a timely manner, of contact tracing of suspected carriers, and of containing the spread by limiting the movement of people within and between regions. Valuable lessons have been learnt but at considerable cost. It is appropriate to quote Bill Gates from his 2015 TED talk that was also published in the New England Journal of Medicine (75): *“*Perhaps the only good news from the tragic Ebola epidemic in Guinea, Sierra Leone, and Liberia is that it may serve as a wake-up call: we must prepare for future epidemics of diseases that may spread.” Hopefully, in 2020, the lessons learnt from COVID-19 will stimulate a collaborative global effort and a greater focus on how to better control future pandemics and the development of new drugs and vaccines to target potential zoonotic diseases.
